# Supplementary figures and images for: The rise of obfuscated Android malware and impacts on detection methods
Source: PeerJ Comput Sci. 2022 Mar 9;8:e907. doi: 10.7717/peerj-cs.907 (PMC9044361; doi:10.7717/peerj-cs.907)

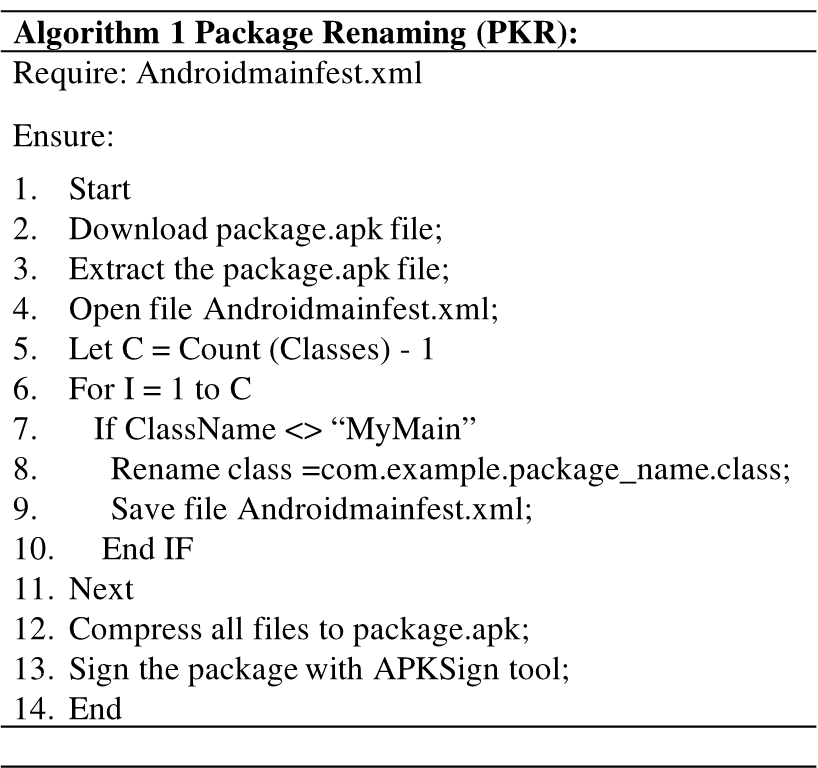

Supplement: Supplemental Information 1 — It is the process of unpacking the APK file and repacking the original application files but signing the APK file with a developer security key. [file peerj-cs-08-907-s001.png]
